# DNA Imprinting and Differentially Expressed Genes in *Longissimus thoracis* Muscle of *Bos indicus* Submitted to Early Weaning Management

**DOI:** 10.3390/epigenomes8040045

**Published:** 2024-12-04

**Authors:** Gustavo Tinoco, Gustavo Russo, Rogério Curi, Marcelo Vicari, Paloma Melo, Isabella Souza, Juliana Torrecilhas, Philipe Moriel, Welder Baldassini, Luis Chardulo, Otávio Neto, Guilherme Pereira

**Affiliations:** 1 School of Agriculture and Veterinary Sciences (FCAV), São Paulo State University (Unesp), Jaboticabal 14884-900, SP, Brazil; gustavo.tinoco@unesp.br (G.T.); gustavo.russo@unesp.br (G.R.); paloma.leandra@unesp.br (P.M.); 2 School of Veterinary and Animal Science (FMVZ), São Paulo State University (Unesp), Botucatu 18618-681, SP, Brazil; rogerio.curi@unesp.br (R.C.); isabella.lissa@unesp.br (I.S.); juliana.akamine@unesp.br (J.T.); w.baldassini@unesp.br (W.B.); luis.artur@unesp.br (L.C.); otavio.machado@unesp.br (O.N.); 3 Department of Structural, Molecular Biology and Genetics, Ponta Grossa State University (Uepg), Ponta Grossa 84030-900, PR, Brazil; vicarimr@uepg.br; 4 Range Cattle Research and Education Center, University of Florida, Ona, FL 33865, USA; pmoriel@ufl.edu

**Keywords:** epigenetics, early weaning, adipogenesis, meat

## Abstract

**Background/Objectives:** Early weaning management followed by energy supplementation can lead to metabolic alterations in the calf that exert long-term effects on the animal’s health and performance. It is believed that the main molecular basis underlying these metabolic adaptations are epigenetic mechanisms that regulate, activate, or silence genes at different stages of development and/or in response to different environmental stimuli. However, little is known about postnatal metabolic programming in *Bos indicus*. Therefore, this study aimed to compare the DNA methylation profile of Nellore animals submitted to conventional and early weaning and to correlate the findings with genes differentially expressed in the *Longissimus thoracis* skeletal muscle of *Bos indicus* cattle. **Methods:** For this, we used Reduced Representation Bisulfite Sequencing (RRBS) and RNA-Sequencing techniques to prospect differentially methylated genes (DMGs). **Results:** A total of 481 differentially methylated regions were identified, with 52% (250) being hypermethylated and 48% (231) hypomethylated. Functional enrichment analysis of 53 differentially methylated and differentially expressed genes was performed. The main enriched terms and pathways were associated with 3′-5′-cyclic adenosine monophosphate (*cAMP*) signaling, which presents the upregulated adenylate cyclase 3 (*ADCY3*) gene and significatively hypomethylated in the promoter region. Alterations in cAMP signaling are involved in numerous processes, many of them related to lipid metabolism. The relative differential expression of key genes of this pathway demonstrates the relationship between cAMP signaling and de novo lipogenesis. **Conclusions**: These findings suggest an important role of postnatal metabolic programming through DNA methylation mechanisms in determining fat deposition in beef.

## 1. Introduction

In cattle, early weaning consists of separating the calves from cows at less than 180 days of age [1] and is aimed at reducing the stress of suckling and nutritional requirements of the cow, thus enabling early body condition recovery and return to estrus [2]. However, attention must be paid to early weaned calves in order to avoid malnutrition [3,4]. Furthermore, energy supplementation of early weaned calves can be used to enhance characteristics of interest in animal production [5,6]. In breeds of European origin, early weaning has been shown to exert a major effect on the physiology of calves destined for slaughter when compared to those conventionally weaned at 210 days of age [6,7].

Studies have shown that early weaned calves supplemented with energy sources can achieve superior performance in systems that employ a slaughter age between 18 and 24 months, such as higher body weight, greater subcutaneous fat thickness, greater *Longissimus thoracis* muscle area, and higher intramuscular fat levels [5,6,8]. Among the key mechanisms underlying these metabolic changes are epigenetic modifications that can affect gene modulation and expression in response to environmental and nutritional stimuli [9] and, consequently, phenotypes.

The origin of these metabolic modifications is in the regulation of complex signaling pathways related to muscle growth and adipogenesis, for example [10]. The activation of satellite cells, important in postnatal muscle growth and regeneration, is regulated by epigenetic events [11]. The regulation of MyoD expression is a notable example of the relevance of DNA methylation in the control of skeletal myogenesis. Selective expression of MyoD occurs in skeletal muscle cells, whereas its expression in non-muscle cells is prevented by DNA methylation [12]. According to [13], DNA methylation also regulates adipogenesis through a coordinated control of positive and negative transcription factors. In mammals, postnatal nutrition, especially in the first months of life, influences gene regulation that leads to changes in metabolism and growth, mediated by epigenetic marks [14]. In the context of these mechanisms (DNA methylation, histone protein modifications, and non-coding RNAs), DNA methylation, as the most stable and best-elucidated chemical modification, has been the most studied in cattle in response to diet [15,16]. DNA methylation can alter gene expression by forming a physical barrier that inhibits the binding of general transcription factors or affecting the condensation state of chromatin; in addition, DNA methylation is involved in the recruitment of methyl-binding proteins that form repressive complexes [17,18]. DNA methylation and histone modification, despite being carried out by different chemical reactions and requiring different sets of enzymes, are more closely linked and there appears to be a relationship where one influences the other [19]. Studies have also reported the involvement of histone alterations [20,21] and ncRNA abundance [22,23] in response to diet during postnatal development. Diet is known to play a key role in determining the DNA methylation pattern by providing substrates required for methylation and cofactors that modulate the enzymatic activity of DNA methyltransferases, as well as the activity of enzymes that regulate one-carbon metabolism [24]. S-adenosylmethionine (SAM), which is synthesized in the methionine cycle from various dietary precursors, is a potent aminopropyl and methyl donor within the one-carbon cycle and serves as the main substrate for the methylation of DNA, associated proteins, and RNA [25,26].

Epigenetic programming is sensitive to nutritional factors, especially during the prenatal period and early phases of life of animals [24,27]. Thus, nutritional alterations can induce “metabolic imprinting”, a mechanism whereby adequate nutrition in the first months after birth alters glucose metabolism and causes long-term beneficial effects on the health and performance of animals [28]. This mechanism proposes critical periods of development, known as “nutritional windows”, in which changes in the DNA methylation pattern and gene expression can permanently modify the animal’s metabolism [14].

At present, there are no omics studies that have associated epigenetics, particularly DNA methylation, with gene expression in Nellore cattle submitted to early weaning and energy supplementation. In this context, we examined the effects of distinct nutritional management protocols on DNA methylation patterns in the *Longissimus thoracis* muscle of Nelore calves during the perinatal phase, and how these epigenetic modifications may have influenced the gene expression profile.

## 2. Results

### 2.1. Reduced-Representation Bisulfite Sequencing

A mean number of 30 million 150-bp reads were generated for the EW group and 28 million 150-bp reads for the CW group. After quality control and adapter removal, the raw number of reads decreased by approximately 1.6% in both groups, corresponding to the number of clean reads. The overall read mapping rate was 76.45% and 76.55% in the EW and CW groups, respectively (Table 1).

### 2.2. DNA Methylation Patterns in Muscle

A mean percentage of 3.84% mCpG, 0.54% mCHG, and 0.47% mCHH was detected in the genome of EW animals, while these percentages were 3.79%, 0.51%, and 0.44%, respectively, in the CW group (Table 2).

**Figure 1:**
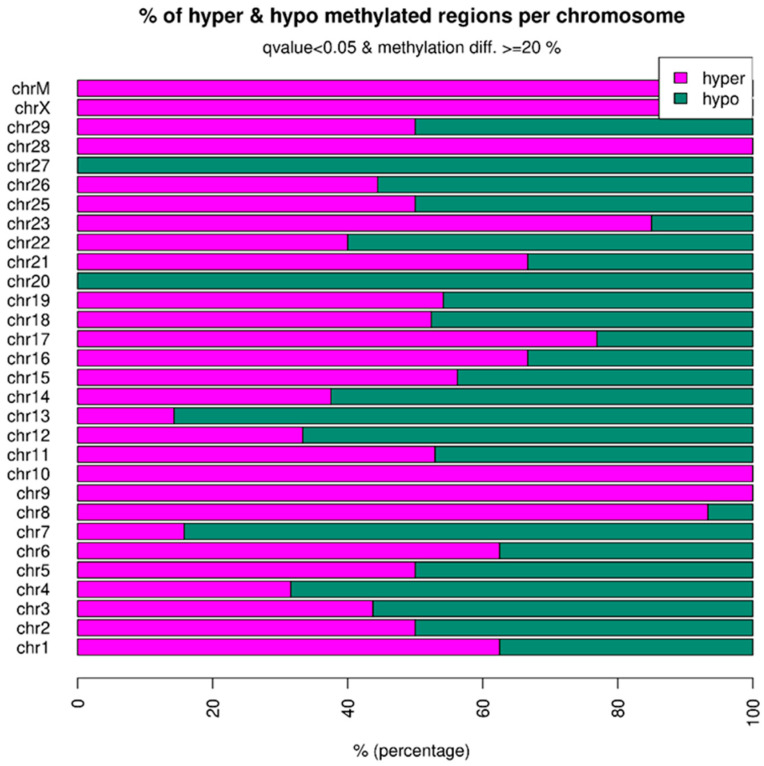
Percentage of hyper- and hypomethylated regions per chromosome of the reference genome (*Bos taurus* ARS-UCD1.2).

We found 481 DMRs showing differences in methylation between the two weaning groups at 205 days (q-value < 0.05 and methylation difference ≥ 20%) (Supplementary Table S1). Balanced ratios of hypo- and hypermethylated regions were observed in the two groups (CW vs. EW). However, there was a slightly higher level of methylation in early weaned animals, with approximately 52% (250) of hypermethylated regions and 48% (231) of hypomethylated regions (Figure 1).

### 2.3. Distribution of Differentially Methylated Regions Across the Genome

**Figure 2:**
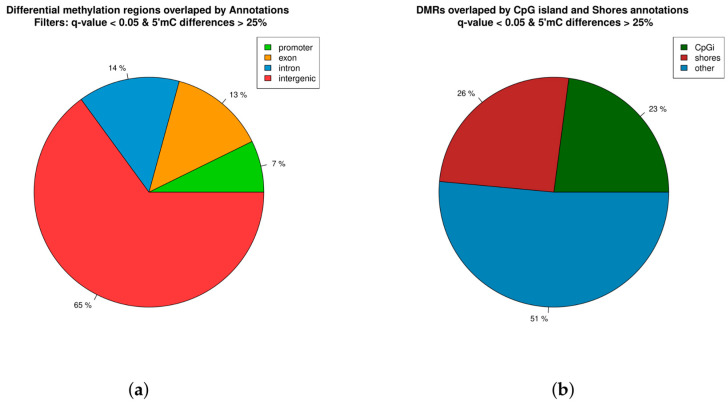
Proportion of differentially methylated regions (DMRs) overlapping intergenic regions, gene body (exon and intron), and promoter regions (**a**). Proportion of DMRs in CpG islands (CpGi), shores, and others (**b**).

Regarding functional location, DMRs were most frequently present in intergenic regions (65%), followed by introns (14%), exons (13%), and promoters/TSSes (7%) (Figure 2a). Approximately 23% of DMRs were located in CpG islands, while 26% were present in shores (Figure 2b; Supplementary Table S1).

There were 172 DMRs overlapping 53 genes, some of them in more than one window and at different functional locations (Supplementary Table S2). Of these prospected regions, 80 DMRs were hypermethylated in the EW group, while the other 92 were hypomethylated. These DMRs overlapped 53 different genes, which were classified as DMGs. Regarding functional location, 16 DMRs were located in gene promoters, while three others were present in TSSes (Table 3).

### 2.4. Functional Enrichment Analysis

Functional enrichment analysis considering the 53 DMGs/DEGs between the EW and CW groups highlighted the following biological processes related to 3′-5′-cyclic adenosine monophosphate (cAMP) signaling and positive lipid regulation: cAMP metabolic process (GO:0046058), cAMP biosynthetic process (GO:0006171), and positive regulation of lipid metabolic process (GO:0045834). Regarding metabolic pathways (KEGG), growth-related pathways can be highlighted, such as growth hormone synthesis, secretion and action, GnRH signaling, and others (Table 4).

### 2.5. Relationship Between Methylation and Gene Expression

**Figure 3:**
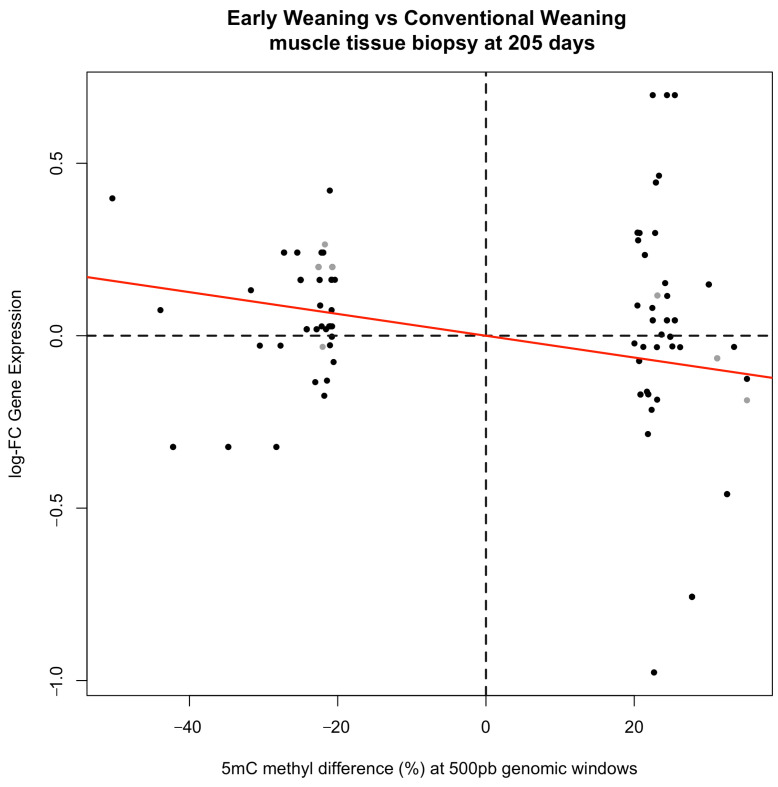
Scatter plot of methylation percentage and gene expression level in the early weaning vs. conventional weaning groups at 205 days. Gray dots indicate promoter regions. The black dots represent gene body and intergenic regions. The red line is the estimated regression line.

In general, a trend can be seen towards a relationship between gene expression and levels of intragenic DNA methylation or methylation in promoter regions of the respective genes (Figure 3, Supplementary Table S3). The estimated regression coefficient was −0.00316, with a *p*-value of 0.0002. The significant slope indicates that many genes harbor hypermethylated regions associated with a decline in expression and hypomethylated regions associated with high gene expression. However, different associations have been reported in the literature in which highly methylated gene bodies tend to exhibit high transcription activity [29]. The *ADCY3* (adenylate cyclase 3), *STUB1* (STIP1 homology and U-box containing protein 1), and *ZNF557* (zinc finger protein 557) genes contained low proportions of 5mC sites in the promoter regions of genes upregulated in the EW group. The same was observed in the CW group for the *TECR* (trans-2,3-enoyl-CoA reductase), *GMDS* (GDP-mannose 4,6-dehydratase), *SSPO* (SCO-spondin), *SULF2* (sulfatase 2), *MACROD1* (mono-ADP ribosylhydrolase 1), *ARID3B* (AT-rich interaction domain 3B), and *ARHGEF10L* (rho guanine nucleotide exchange factor 10 like) genes (Supplementary Table S3).

Since not all genes involved in a pathway have their regulation mediated by DNA methylation but can be affected by genes regulated by these mechanisms, the DEG data are important for elucidating the activity of metabolic pathways. These results are therefore essential for studies on epigenetic regulation by DNA methylation and are presented in Supplementary Table S4.

## 3. Discussion

In the present study, the comparison of groups of Nellore calves submitted to EW and energy supplementation and calves submitted to CW revealed an important correlation between methylated DNA regions and gene expression, with many of the genes being associated with fat metabolism. It is known that the epigenome is sensitive to nutritional factors [30]. A comparison of the EW vs. CW groups showed a low percent variation in methylation. This finding can be explained by the conserved methylation profile of cattle [30], as well as by the fact that the animals were exposed to the same management conditions from birth to 120 days of age. However, the exposure of EW animals to a period of “nutritional window” (121 to 205 days) under energy supplementation exerted an epigenetic effect, with the identification of 485 DMRs. This finding might be related to the presence of so-called epigenetic modifiers—molecules that are important methyl donors and/or cofactors in DNA methylation reactions such as methionine, choline, folate, and vitamin B12 [31]. Thus, the hypermethylation of genes during the postnatal metabolic programming that occurred in the EW group may be the result of nutritional factors. Since these animals received a high-protein diet (20%), the availability of the compounds mentioned above, which are recognized as methyl donors for DNA methylation, was greater. These compounds enter one-carbon metabolism at different sites but are ultimately converted to the methyl group donor SAM, which provides one methyl group for DNA methylation [32]. In one-carbon metabolism, most methyl groups are derived from choline (60%), followed by methionine (20%) and folate (10–20%) [33]. Methionine is an essential amino acid that is regenerated from homocysteine. In addition to its importance for protein synthesis, when converted to SAM, methionine participates in more than 100 methylation reactions in the body [34]. Thus, variations in dietary methionine may affect DNA methylation [35]. However, some studies suggest that there is no simple correlation between the concentration of methyl donors and DNA methylation and that complex mechanisms of competitive inhibition of DNA methyltransferases may contribute to DNA imprinting [24,36]. DNA demethylation can occur passively when there is an absence of maintenance methyltransferases during DNA replication, or actively, involving the Ten-Eleven translocation (TET) protein family. Through three consecutive oxidative reactions, TET proteins are responsible for the conversion of 5-methylcytosine (5-mC) into three intermediate products: 5-hydroxymethylcytosine (5-hmC), 5-formylcytosine (5-fC), and 5-carboxylcytosine (5-caC) [37]. Currently, 5-hmC is well accepted as the main intermediate in DNA demethylation, where it also plays a role in genome regulation. Because of this, it has been the target of many studies that aim to elucidate its biological role. Although its concentration is very low in relation to 5′mC, it presents high variability in its levels and has already been detected in several tissues and cells. Regarding its locations in the genome, it has been described in enhancers, CpG island promoters, and in the gene body. In CpGi, 5′hmC plays a crucial role in maintaining promoters in an unmethylated state, while in the gene body, 5′hmC is thought to inhibit the initiation of antisense transcription [38]. The RRBS technique used in our study does not allow distinguishing between 5-mC and 5-hmC; therefore, future studies involving capture methods based on immunoaffinity, protein affinity, and enzymatic modification or chemical labeling [39] may benefit from incorporating research on 5-hmC. Furthermore, to have a more detailed understanding of postnatal metabolic changes influenced by diet, other epigenetic mechanisms (in addition to DNA methylation) can be the target of future studies that will certainly produce results that improve our biological understanding.

*Bos indicus* breeds are known for their lower meat tenderness and intramuscular fat deposition when compared to *Bos taurus* [40]. Thus, the identification of gene pathways that could improve fat deposition and meat tenderness in *Bos indicus* is an important strategy for this animal production sector [40]. Within this context, studies of European cattle breeds suggested that early weaning management leads to changes in DNA methylation patterns and in the expression of genes involved in metabolism [6,8]. In the study by [6], metabolic programming due to early weaning resulted in significant changes in carcass fat deposition, suggesting that early postnatal metabolic imprinting events can be explored as a management tool to improve the value and quality of meat based on increased marbling. In our study, EW animals exhibited 53 DMGs/DEGs. These results are consistent with the effects of early postnatal metabolic programming on the expression of gene levels involved in numerous biological processes, including those related to the positive regulation of lipid metabolic processes.

Among the main genes exhibiting significant epigenetic changes and alteration in expression due to early weaning, we highlight the *ADCY3* and *AKT1* genes. The *ADCY3* gene is involved in the regulation of different processes, including embryogenesis, hormone secretion, glycogen degradation, smooth muscle relaxation, cardiac muscle contraction, and olfaction [41,42,43]. In general, adenylyl cyclases (ADCYs) are enzymes that catalyze the synthesis of cAMP from adenosine triphosphate (ATP) [44]. The present results showed that the cAMP signaling pathway was enriched in the EW group. Cyclic AMP is a second messenger involved in intracellular signal transduction that is associated with the function of kinases in different biochemical processes, including the regulation of carbohydrate and lipid metabolism [44,45]. Increases in cAMP ultimately activate a number of enzymes and transcription factors that regulate satiety and metabolism [46].

In this scenario, we evaluated some pathways linked to energy metabolism and regulated by overexpression of *ADCY3* regarding genes differentially expressed in the EW group. The altered expression of *ADCY3* is related to an increase in protein kinase cAMP-activated catalytic subunit alpha (PRKACA). This kinase signal changes in adipogenesis, thermogenesis, fatty acid oxidation, and insulin resistance pathways [42]. The evaluation of DEGs in this study did not provide solid data on changes in adipogenesis, thermogenesis, or fatty acid oxidation; however, genes involved in de novo lipogenesis were upregulated.

Adipogenesis consists of the differentiation of mesenchymal stem cells into preadipocytes and the proliferation of these cells, the differentiation of preadipocytes into adipocytes, and the conversion to lipid-assimilating cells [47,48,49]. The mechanisms that control adipogenesis in skeletal muscle are largely regulated by transcription factors of the enhancer binding protein (CEBP) and peroxisome proliferator-activated receptor γ (*PPARg*) family [49,50,51]. The *CEBP* and *PPARg* genes were found to be overexpressed but not differentially methylated in the EW group. However, various other genes that are important for adipogenesis did not show consistent changes in expression, including bone morphogenetic protein 4 (*BMP4*), PR domain-containing protein 16 (*PRDM16*), fibroblast growth factor 10 (*FGF10*), and fatty acid binding protein 4 adipocyte (*FABP4*). For example, *PRDM16* acts as a switch between the myogenic lineage and brown adipocytes [52,53]. Cells expressing *PRDM16* do not undergo differentiation to the myogenic lineage. Thus, although the EW group exhibited a significant change in *ADCY3*, the alteration in the expression of genes involved in adipogenesis was less consistent. In fact, it has been proposed that adipogenesis starts in the second half of gestation in ruminants and reaches its highest activity during the perinatal period [49]. In the present study, the postnatal period was the nutritional window, i.e., after the main phase of adipocyte differentiation.

Beta-oxidation and thermogenesis are other processes that can be influenced by excess ADCY3 and PRKACA [42]. Although the expression of these regulators was altered in the EW group, no significant changes were observed in the expression of the hormone-sensitive lipase (HSL or LIPE) or carnitine palmitoyltransferase (*CPT1* and *CPT2*) gene, indicating the lack of a difference in beta-oxidation between groups. Cyclic AMP-responsive element-binding protein 1 (*CREB1*) is regulated by PRKACA during thermogenesis. Likewise, neither the expression of *CREB1* nor of other genes involved in thermogenesis was significantly altered in the EW group.

The insulin resistance pathway has also been suggested to respond to cAMP signals [42]. According to [6], an increase in gluconeogenic substrates for ruminal fermentation and in the concentration of insulin in response to circulating glucose leads to greater cellular uptake and utilization of glucose for fat deposition in intramuscular adipocytes proliferated by imprinting, increasing marbling during the growth phase. The gene expression data obtained for the EW group showed increased expression of protein kinase AMP-activated catalytic subunit alpha 1 (PRKAA1 or AMPK), an important mediator of the insulin resistance pathway, along with phosphatidylinositol-4,5-bisphosphate 3-kinase catalytic subunit beta (PI3KCB) and AKT serine/threonine kinase 1 (AKT1) [42]. AKT1 is responsible for the regulation of glucose uptake by mediating the insulin-induced translocation of solute carrier family 2 member 4 or glucose transporter type 4 (SLC2A4 or GLUT4) to the cell surface [54]. *AKT1* was hypermethylated in the promoter and intron regions in the EW group; however, no significant alteration in gene expression was observed. Furthermore, the analysis of DEGs in this study revealed no alteration.

AKT signaling has been reported to prevent the degradation of sterol regulatory element binding transcription factor 1 (*SREBP1*) and thus to promote de novo lipid synthesis [55]. This gene belongs to a family of transcription factors [55] and encodes an important transcriptional regulator of genes involved in lipid synthesis and metabolism, including fatty acid synthase (*FASN*) and ATP-citrate lyase (*ACLY*) [56,57]. *SREBP1* was overexpressed, demonstrating that *AKT1* hypermethylation did not compromise gene expression. Other factors may be involved in the positive regulation of de novo lipid synthesis in the EW group.

Indeed, the DEG data demonstrated overexpression of genes involved in the de novo lipid synthesis pathway, including acetyl-CoA synthetase (*ACSS2*), *ACLY*, acetyl-CoA carboxylase (*ACACA*), stearoyl-CoA desaturase (*SCD*), and *FASN*, as well as the transcription factor SREBF-1, as also reported by [58]. An intriguing question raised here is how the postnatal metabolic programming performed in this study has stimulated lipogenesis. According to the review by [49], fat synthesis requires the incorporation of triglycerides into the adipose tissue of the animal after absorption of dietary fatty acids or de novo synthesis of other fatty acids. Acetate—a volatile fatty acid produced during ruminal fermentation—is the main precursor molecule for the synthesis of fatty acids in ruminants [59]. After ruminal absorption and already in the adipocyte, acetyl-CoA synthetase (ACSS2) converts acetate into acetyl-CoA [60], which, in the presence of reduced nicotinamide adenine dinucleotide phosphate (NADPH), is crucial for de novo fatty acid synthesis [49].

Interestingly, the cAMP signaling pathway enriched in this study regulated various other processes through ADCYs. One such process involves the sodium/potassium-transporting ATPase subunits gamma, alpha-1, and beta-2 (KEGG *Bos taurus*: 281773). These proteins, in turn, participate in digestion and absorption pathways (bta04973 and bta04974) in the intestinal epithelium, including acetate absorption in colonic epithelial cells. Although the expression of ATPases was not obtained for intestinal cells in this study, indirect evidence of increased acetate absorption and de novo lipid synthesis stems from the overexpression of *FABP4*. Expressed mainly in adipocytes, the product of the *FABP4* gene is a cytoplasmic protein that binds to long-chain fatty acids and other hydrophobic ligands and plays a role in uptake, transport, and hydrolysis [61,62]. Increased expression of this gene has been associated with greater intramuscular fat deposition, carcass weight, and fatty acid composition in beef cattle [58,63,64]. Further investigations may address the direct impact of cAMP and ADCY3 signaling on metabolic pathways in the liver, adipose tissue, and muscle, elucidating the specific mechanisms and influence of this pathway on growth and metabolic adaptation in post-weaning calves. It is described in the literature that the cAMP pathway and its association with ADCY3 affect several cellular events and signaling molecules, and are involved in the regulation of adipogenesis, lipogenesis, and lipolysis in adipocytes, thus possibly also regulating feed efficiency [65,66]. Studies exploring this area may be beneficial to improve productivity in cattle.

Taken together, the data reinforce the proposals of greater fat deposition in cattle submitted to early weaning [6,8,58]. Furthermore, emerging evidence indicates that postnatal metabolic programming through epigenetic modification mainly exerts an effect on the ADCY*3* receptor which, in turn, leads to numerous changes in pathways linked to cAMP signaling.

## 4. Materials and Methods

### 4.1. Animals and Collection of Muscle Tissue

The field experiment was conducted in the town of Cáceres, State of Mato Grosso, Brazil. The climate of the region is characterized as tropical, with two well-defined seasons (dry in winter and wet in summer), an average annual temperature of 25 °C, and an average precipitation of 1396 mm/year. Forty purebred Nellore cows that met the following criteria were selected from a herd of 3500 animals: same age, same number of calvings, and having given birth to male calves. In addition, cows with a maximum difference between calvings of 15 days were chosen.

These cows and their calves were kept in a rotational grazing system of *Brachiaria decumbens* paddocks, with minerals being available ad libitum. At 120 days after the average calving date, 20 calves were weaned and started to receive a concentrate-based supplementation in a new paddock consisting of *Brachiaria decumbens* where they remained until 205 days of age. These calves formed the early weaning (EW) group. The other 20 calves remained with the cows until 205 days of age when they were weaned conventionally (CW group) (Table 5). Thus, except for the period between 120 and 205 days corresponding to the different treatments, the animals shared a common environment and received the same management and diet.

For molecular analyses, biopsies of *Longissimus thoracis* muscle performed between the 12th and 13th rib were obtained from 40 calves (20 EW and 20 CW) at 205 days of age. The collected biopsies were kept in liquid nitrogen and transported to the Laboratory of Genetics and Animal Breeding (LGMA) of the School of Veterinary Medicine and Animal Science (FMVZ), São Paulo State University (Unesp), Botucatu. These samples were stored frozen at −80 °C until the time of RNA and DNA extraction.

### 4.2. Genomic DNA Extraction, RRBS, Quality Control and Alignment to Reference Genome

For DNA methylation analysis, total genomic DNA was extracted from the muscle biopsies of five individuals from each group (EW and CW) for reduced-representation bisulfite sequencing (RRBS), which focuses on genomic regions rich in CpG. The animals were selected in a way to avoid biased sampling; therefore, they were sampled randomly. For this purpose, 10 genomic libraries were prepared and sequenced (five EW vs. five CW) by Zimo Research (Irvine, CA, USA), using 400 ng of initial input genomic DNA, which was digested with 30 units of the MspI restriction enzyme to digest CpG-rich regions. Fragments were ligated to pre-annealed adapters containing 5-methylcytosine instead of cytosine according to Illumina’s specified guidelines. Adaptor-ligated fragments ≥ 50 bp in size were recovered using the DNA Clean & ConcentratorTM-5 (Cat#: D4003). The fragments were then bisulfite-treated using the EZ DNA Methylation-LightningTM Kit (Cat#: D5030). Preparative-scale PCR was performed, and the resulting products were purified with DNA Clean & ConcentratorTM-5 (Cat#: D4003) for sequencing on an Illumina NovaSeq 6000 platform (150 bp PE reads).

The reads obtained were identified using standard base-calling software (Illumina, San Diego, CA, USA) and the quality of the raw fastq files was analyzed with FastQC 0.11.9 [67]. Adapters were trimmed, and low-quality reads were removed using the TrimGalore 0.6.4 software [68]. Alignment to the reference genome (*Bos taurus* ARS-UCD1.2) was performed using the Bismark 0.22.3 software [69]. Total methylated (C) and unmethylated (T) cytosine reads were called using the MethylDackel v0.5.0 tool (https://github.com/dpryan79/MethylDackel (accessed on 17 May 2023)). The minimum depth of CpG sites of 10× and the percentage of genome coverage of regions with high CpG density (approximately 10% of the total CpG sites), around 6Gb per sample, and the insert size (150 bp) were chosen based on the best performance of the RRBS technique described for ruminants (sheep and cattle) described by [70,71].

### 4.3. Identification and Characterization of Differentially Methylated Regions and Genes

First, to better understand the context of cytosine methylation, metrics that distinguish cytosines directly linked to guanines (CpG), indirectly linked to guanines (CHG), or not linked to guanines (CHH) were obtained with the MethylDackel v0.5.0 tool, in which H is the IUPAC ambiguity code for any nucleotide other than G. In addition, coverage metrics, correlation of methylation levels between samples, and chromosomal distribution of methylated cytosines were generated with the R package methylKit [72].

To identify differential DNA methylation patterns, the methylKit v1.24.0 package [72] was used for filtering, discarding bases with read coverage < 10×, and normalization of T/C ratios across samples. In addition, for each sample, we used a sliding window algorithm based on the defined window size and step size. The genome was divided into multiple fragments with a window size of 1000 bp and overlapping regions of 500 bp, which allowed the calculation of the T/C ratios per delimited and overlapping genomic regions. Based on Fisher’s exact test, it was then possible to identify positions containing differentially methylated cytosines (DMCs) between groups, as well as genomic regions containing average proportions of DMCs between groups (DMRs). For a more robust identification of DMCs and DMRs, in addition to the *p*-value adjusted for multiple comparisons (q-value < 0.05), the minimum difference of methylated cytosines of 20% between groups was also used in both cases (DMCs and DMRs).

For the description of the basic DMR annotation, the plotTargetAnnotation function was applied through the interface of the generation [73] and methylKit [72] packages of R [74], in which DMRs can overlap with only promoter regions, intergenic regions, exons, and introns. Overlaps with CpG islands and shores are also observed.

For the subsequent gene expression and methylation combined analyses, which require a greater amount of genomic information in terms of DMR annotation, were performed a customized annotation script using NCBI RefSeq and CpG_island files obtained from the UCSC Genome Browser (https://genome.ucsc.edu/cgi-bin/hgTables (accessed on 20 March 2023)) based on the URS-ACD1.2 assembly. In this case, in addition to the information obtained above, the position of introns and exons was discriminated, as well as the overlap between CpG islands and shores in gene and promoter regions. The promoter region was defined as 2000 bp downstream and 500 bp upstream of the transcription start site (TSS).

### 4.4. Relative Gene Expression

To access the expression level of the genes studied here, a transcriptome dataset of *Longissimus thoracis* muscle obtained for the same samples as used in this experiment was employed, which are available at https://drive.google.com/drive/folders/113q1QKulYBU22EC7ZwtbWk-2ORFIEzek?usp=share_link (accessed on 16 January 2024). The data obtained from the n x m matrix containing the genes symbol (n) and the number of samples (m) were normalized using counts per million (CPM) to obtain the relative expression between treatments (CW and EW), given as the logarithmic function of fold change (logFC). The significance values of the difference between mean treatment counts were obtained with a generalized linear model assuming a negative binomial distribution, followed by the application of the Quasi Likelihood F-test. For this purpose, the common dispersion parameter was estimated, and normalized Trimmed Mean of M-values (TMM) data were obtained considering the relative size of the libraries. The edgeR v. 4.2.0 package [75] in R v. 4.3.3 was used for these differential expression analyses [74].

### 4.5. Relationship Between Differentially Methylated Genes and Genes Expression

To investigate the relationship between differentially methylated genes (DMGs) and their expression levels, a graphical representation of a scatter plot and statistical associations between methylation percentage and gene expression level was performed. In general, the positions and relative methylation levels (hyper- or hypomethylated) of genomic regions to which these DMRs were mapped were analyzed in relation to the level of gene expression (over- or underexpressed). Next, the correlation between the gene expression level given as logFC of DMGs and the methylation percentage of the DMRs was analyzed using Pearson’s correlation coefficient. No multiple testing corrections were applied. For linear regression analysis, the assumptions of homogeneity of variances and normality of residuals were checked. Finally, a trend line was fitted by simple linear regression in which the level of gene expression given as logFC was the response variable, and the methylation level expressed as a percentage was the explanatory variable. For this purpose, only DMGs that were also differentially expressed were considered.

### 4.6. Functional Enrichment of Differentially Methylated Genes

Enrichment analysis of gene ontology biological processes (GO_BP) and metabolic pathways (KEGG) was performed using the EnrichR v. 3.2 software [76], considering differentially methylated genes (DMGs) that were also differentially expressed (DEGs) obtained by differential gene expression analysis [44]. The Benjamini–Hochberg method was used to correct multiple hypotheses. GO terms and KEGG pathways with a *p*-value < 0.01 and q-value < 0.1 were considered to be significantly enriched.

## 5. Conclusions

The data obtained in this study suggest that early weaning of Nellore cattle alters the methylation profile of some genes linked to metabolism. The *ADCY3* gene is one of the main genes that were differentially methylated and differentially expressed. Functional enrichment analysis suggested that the cAMP signaling pathway may play an important role in the metabolic changes observed in early weaned cattle. Cyclic AMP signaling is involved in adipogenesis and lipogenesis processes and the DEG data found here demonstrated strong alteration in the expression of genes involved in de novo lipogenesis. The differential methylation of metabolism genes due to early weaning supplementation found in this study suggests that *ADCY3* may be one of the key genes for understanding metabolic programming associated with fat deposition pathways in cattle and indicates that epigenetic mechanisms such as DNA methylation may be involved in the energetic metabolism adaptation caused by diet and environmental modification in Nellore calves early weaned.

## Figures and Tables

**Table 1:** Descriptive statistics of reduced-representation bisulfite sequencing.

	Early Weaning	Conventional Weaning
Number of raw reads	30,703,133	28,494,972
Number of clean reads	30,194,010	28,023,825
Number of aligned reads	23,084,052	21,452,788
Percentage of aligned reads	76.45%	76.55%

**Table 2:** Percentage of methylated CpG, CHG, and CHH in the genome of early and conventionally weaned animals.

	Early Weaning	Conventional Weaning
mCpG (%)	3.84%	3.79%
mCHG (%)	0.54%	0.51%
mCHH (%)	0.47%	0.44%

**Table 3:** Differentially methylated regions (DMRs) located in promotors and transcription start sites (TSSes).

DMG	Description	LocChr	Start	End	DMR. *p*-Value	Meth.diff	Category
*ADCY3*	adenylate cyclase 3	chr11	74335201	74335700	2.83 × 10^−24^	−20.7252	TSSes
*ADCY3*		chr11	74335301	74335800	1.92 × 10^−24^	−22.5866	TSSes
*ADCY3*		chr11	74335201	74335700	2.83 × 10^−24^	−20.7252	promoters
*ADCY3*		chr11	74335301	74335800	1.92 × 10^−24^	−22.5866	promoters
*AKT1*	AKT serine/threonine kinase 1	chr21	69246101	69246600	3.72 × 10^−15^	31.18985	promoters
*ATP6*	mitochondrially encoded ATP synthase 6	chrM	6201	6700	5.75 × 10^−23^	35.20596	promoters
*COX1*	cytochrome c oxidase subunit I	chrM	6201	6700	5.75 × 10^−23^	35.20596	promoters
*F2*	coagulation factor II, thrombin	chr15	76656801	76657300	2.77 × 10^−6^	21.88917	promoters
*F2*		chr15	76656801	76657300	2.77 × 10^−6^	21.88917	TSSes
*MAP2K1*	mitogen-activated protein kinase 1	chr10	13275401	13275900	5.80 × 10^−5^	23.12619	promoters
*MAP2K1*		chr10	13275201	13275700	5.80 × 10^−5^	23.12619	promoters
*MAP2K1*		chr10	13275301	13275800	5.80 × 10^−5^	23.12619	promoters
*MRPL28*	mitochondrial ribosomal protein L28	chr25	363101	363600	0.000182	−20.7854	promoters
*RUVBL1*	RuvB like AAA ATPase 1	chr22	59508001	59508500	2.67 × 10^−8^	−22.0339	promoters
*STUB1*	STIP1 homology and U-box containing protein 1	chr25	585001	585500	1.74 × 10^−5^	−25	promoters
*STUB1*		chr25	584901	585400	1.74 × 10^−5^	−25	promoters
*STUB1*		chr25	584801	585300	0.000112	−22.4395	promoters
*STUB1*		chr25	585101	585600	1.74 × 10^−5^	−25	promoters
*ZNF557*	zinc finger protein 557	chr7	15991901	15992400	2.77 × 10^−8^	−21.7254	promoters

**Table 4:** GO_BP terms and KEGG pathways enriched for the 53 differentially expressed and methylated genes and their *p*-value and q-value.

Term	Library	*p*-Value	q-Value	Genes
Growth hormone synthesis, secretion and action	KEGG_2021_Human	0.00001	0.00162	*MAP2K1*, *ADCY3*, *ADCY5*, *MAP2K6*, *AKT1*
Rap1 signaling pathway	KEGG_2021_Human	0.00001	0.00162	*ADCY3*, *MAP2K6*, *ADCY5*, *PGF*, *MAP2K1*, *AKT1*
Positive regulation of lipid metabolic process (GO:0045834)	GO_Biological_Process_2021	0.00003	0.02894	*F2*, *AKT1*, *PPARD*
Phospholipase D signaling pathway	KEGG_2021_Human	0.00004	0.00254	*AKT1*, *ADCY3*, *ADCY5*, *F2*, *MAP2K1*
Pathways in cancer	KEGG_2021_Human	0.00007	0.00310	*MAP2K1*, *PGF*, *ADCY3*, *AKT1*, *ADCY5*, *F2*, *PPARD*, *HEYL*
GnRH signaling pathway	KEGG_2021_Human	0.00010	0.00323	*MAP2K1*, *ADCY3*, *MAP2K6*, *ADCY5*
Connective tissue development (GO:0061448)	GO_Biological_Process_2021	0.00013	0.70289	*COL11A2*, *SULF2*, *DYRK1B*
Progesterone-mediated oocyte maturation	KEGG_2021_Human	0.00014	0.00323	*MAP2K1*, *ADCY3*, *ADCY5*, *AKT1*
Surfactant homeostasis (GO:0043129)	GO_Biological_Process_2021	0.00014	0.02894	*CTSH*, *ABCA3*
Chemical homeostasis within a tissue (GO:0048875)	GO_Biological_Process_2021	0.00014	0.02894	*ABCA3*, *CTSH*
Chemokine signaling pathway	KEGG_2021_Human	0.00015	0.00323	*MAP2K1*, *FGR*, *ADCY3*, *AKT1*, *ADCY5*
Longevity regulating pathway	KEGG_2021_Human	0.00015	0.00323	*ADCY3*, *AKT1*, *ADCY5*, *EHMT1*
Parathyroid hormone synthesis, secretion and action	KEGG_2021_Human	0.00017	0.00323	*ADCY3*, *ADCY5*, *MAP2K1*, *PDE4A*
Focal adhesion	KEGG_2021_Human	0.00018	0.00323	*PGF*, *AKT1*, *ITGB5*, *TNXB*, *MAP2K1*
Cellular response to forskolin (GO:1904322)	GO_Biological_Process_2021	0.00024	0.03297	*ADCY3*, *ADCY5*
Response to forskolin (GO:1904321)	GO_Biological_Process_2021	0.00024	0.03297	*ADCY3*, *ADCY5*
cAMP biosynthetic process (GO:0006171)	GO_Biological_Process_2021	0.00030	0.03526	*ADCY3*, *ADCY5*
Regulation of proteolysis (GO:0030162)	GO_Biological_Process_2021	0.00039	0.03996	*F2*, *AKT1*, *STUB1*
Peptidyl-threonine phosphorylation (GO:0018107)	GO_Biological_Process_2021	0.00054	0.04846	*DYRK1B*, *AKT1*, *MAP2K1*
cAMP metabolic process (GO:0046058)	GO_Biological_Process_2021	0.00061	0.04958	*ADCY3*, *ADCY5*

**Table 5:** Supplementation and diets consisting of natural forage, soybean meal, corn, additives, and minerals supplied to Nellore cattle at different stages of development and submitted to different weaning protocols.

Rearing Phase	Early Weaning	Conventional Weaning
Birth to 120 days	Conventional suckling on *Brachiaria* pasture	
120 to 205 days	Brachiaria decumbens pasture + concentrate-based diet (20% CP ^1^; 75% TDN ^2^): 1 g of DM ^3^/kg BW ^4^	Conventional suckling + *Brachiaria decumbens*

^1^ crude protein; ^2^ total digestible nutrients; ^3^ dry matter; ⁴ body weight.

## Data Availability

The data presented in this study are available on request from the corresponding author.

## Supplementary Materials

The following supporting information can be downloaded at: https://www.mdpi.com/article/10.3390/epigenomes8040045/s1, Table S1: DMR Complete Annotation Methylated Regions; Table S2: DMG Results; Table S3: DMG expression level; Table S4: Relative expression results from edgeR.
